# MicroRNA-148a deficiency promotes hepatic lipid metabolism and hepatocarcinogenesis in mice

**DOI:** 10.1038/cddis.2017.309

**Published:** 2017-07-13

**Authors:** Li Cheng, Yahui Zhu, Han Han, Qiang Zhang, Kaisa Cui, Hongxing Shen, Jinxiang Zhang, Jun Yan, Edward Prochownik, Youjun Li

**Affiliations:** 1 Hubei Key Laboratory of Cell Homeostasis, College of Life Sciences, Wuhan University, Wuhan 430072, China; 2 Medical Research Institute, School of Medicine, Wuhan University, Wuhan 430071, China; 3 Department of Surgery, Wuhan Union Hospital, Wuhan 430022, China; 4 State Key Laboratory of Pharmaceutical Biotechnology and MOE Key Laboratory of Model Animals for Disease Study, Model Animal Research Center, Nanjing University, Nanjing 210008, China; 5 Collaborative Innovation Center for Genetics and Development, Shanghai 200438, China; 6 Division of Hematology/Oncology, Children's Hospital of Pittsburgh of UPMC and The Department of Microbiology and Molecular Genetics, The University of Pittsburgh Medical Center, Pittsburgh, PA 15224, USA

## Abstract

miRNAs are involved in many physiologic and disease processes by virtue of degrading specific mRNAs or inhibiting their translation. miR-148a has been implicated in the control of tumor growth and cholesterol and triglyceride homeostasis using *in vitro* or *in vivo* gene expression- and silencing-based approaches. Here miR-148a knockout (KO) mice were used to investigate the intrinsic role of miR-148a in liver physiology and hepatocarcinogenesis in mice. miR-148a downregulation was found to be correlated with poor clinical outcomes in hepatocellular carcinoma (HCC) patients. Under regular chow diet (RCD) or high fat diet (HFD), miR-148a deletion significantly accelerated DEN-induced hepatocarcinogenesis in mice. Mechanistically, miR-148a deletion promotes lipid metabolic disorders in mice. Moreover, restoration of miR-148a reversed these defects. Finally, miR-148a was found to directly inhibit several key regulators of hepatocarcinogenesis and lipid metabolism. These findings reveal crucial roles for miR-148a in the hepatic lipid metabolism and hepatocarcinogenesis. They further identify miR-148a as a potential therapeutic target for certain liver diseases, including cancer.

miRNAs are a small class of endogenous non-coding RNAs that promote mRNA degradation or translation inhibition and thus control many physiological and disease processes.^1^ Primary liver cancer is the second most common cause of cancer-related death after lung cancer and hepatocellular carcinoma (HCC) is the most common type of primary liver cancer.^2^ Risk factors for HCC include chronic hepatitis B virus (HBV), hepatitis C virus (HCV) infection, alcoholic liver disease as well as non-alcoholic fatty liver disease and metabolic disorders.^2, 3^ Most HCCs develop in the setting of underlying compromised liver function, such as hepatitis, hepatosteatosis and cirrhosis.^2, 3^

Emerging evidence suggests that miRNAs regulate normal liver development and metabolism whereas miRNA dysregulation is associated with a variety of liver disorders, including HCC.^4, 5, 6, 7, 8, 9^ The universal function of miRNAs in normal liver physiology was addressed in a mouse model lacking Dicer function in hepatocytes as Dicer is an essential enzyme for miRNA processing. Despite the loss of mature miRNAs, initial hepatic function was maintained although, over time, the mice exhibited progressive hepatosteatosis, hepatitis and the spontaneously development of HCC.^4, 5^ These findings suggested that miRNAs have critical roles in hepatocyte survival, metabolism, developmental gene regulation and tumor suppression.^4, 5, 6, 7, 8, 9^

miR-148a, highly expressed in adult liver, has been previously shown to control cholesterol and triglyceride homeostasis and circulating lipoprotein levels,^10, 11, 12^ as well as hepatocytic differentiation and the pathogenesis of HCC.^13, 14, 15, 16, 17, 18, 19, 20^ However, the molecular mechanisms of miR-148a in liver physiology and hepatocarcinogenesis remain poorly understood. To address the physiological roles of miR-148a in the liver, a KO mouse model with a germline deletion of miR-148a was used. Our studies show that miR-148a is crucial for hepatic metabolism. It also possesses an intrinsic tumor suppressor function in the DEN-induced hepatocarcinogenesis model.

## Results

### miR-148a downregulation predicts poor HCC patient clinical outcomes

**Figure 1:**
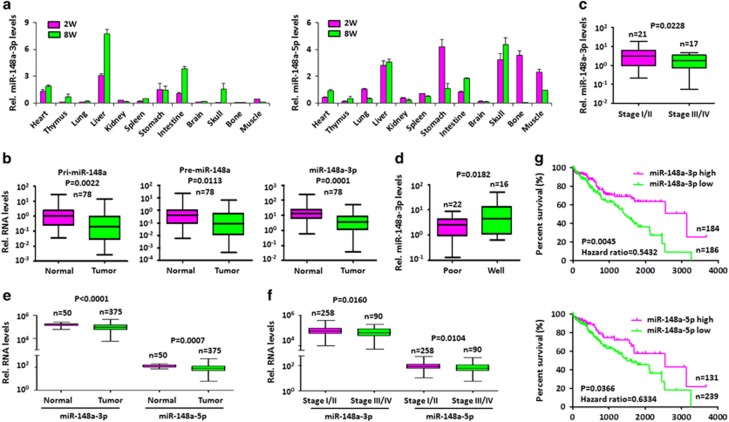
miR-148a is downregulated in HCC, and low expression predicts poor HCC patient outcomes. (**a**) miR-148a expression across mouse tissues of 2-week and 8-week old mice. Data present mean±S.D. (**b)** Expression of Pri/Pre-miR-148a and miR-148a-3p in 78 HCC samples and their adjacent noncancerous tissues. (**a-b**) Pri/Pre-miR-148a and miR-148a-3p RNA levels normalized for U6 were measured by stem-loop RT-qPCR in RNA purified from the above tissues. (**c** and **d**) Association of miR-148a-3p expression with clinical stages (**c**) and tumor differentiation (**d**) in sporadic HCC samples. (**e**) miR-148a-3p/5p expression in normal liver and HCC tissues from TCGA data set. (**f**) Association of miR-148a-3p/5p expression with clinical stages in HCC patients from TCGA data set. (**b–f**) Significance was performed using Wilcoxon signed rank test. The horizontal lines in the box plots represent the median, the boxes represent the interquartile range, and the whiskers represent the minimal and maximal values. (**g**) Survival of human HCC patients from TCGA data set with high versus low miR-148a-3p/5p expression. *P*-values and hazard ratios (HR) are indicated

In our previous studies, miR-148a significantly inhibited HCC cell growth.^20, 21^ The miR-148a locus encodes two miRNAs, miR-148a-3p/5p, which are members of a family of evolutionarily conserved miRNAs that are highly expressed in most mouse tissues, including liver (Figure 1a and Supplementary Figure 1a). To study the clinical significance of miR-148a expression in HCC, Pri/Pre-miR-148a and miR-148a-3p expression levels were measured in total RNA derived from normal hepatocytes HL7702, four HCC cell lines, 78 HCCs and paired normal hepatic tissues using RT-qPCR. The experiments showed that Pri/Pre-miR-148a and miR-148a-3p expression were both significantly lower in HepG2, BEL-7402 and Huh7 compared with HL7702 and FHCC98 (Supplementary Figure 1b). Also Pri/Pre-miR-148a and miR-148a-3p were significantly downregulated in HCC samples compared with paired normal hepatic tissues (Figure 1b). Furthermore, decreased miR-148a expression was significantly associated with advanced stage tumors in HCC patients (Figure 1c). Histologically poorly differentiated HCC also showed a significant association with decreased miR-148a-3p expression relative to well differentiated tumors (Figure 1d). To confirm the above results, the publicly available TCGA datasets of HCC cases were analyzed. In accordance with the above results, miR-148a-3p/5p expression in HCCs was significantly lower than that in normal hepatic tissues (Figure 1e) and the significant association between decreased miR-148a-3p/5p expression and advanced tumor stage was also validated (Figure 1f). To further study the clinical significance of miR-148a expression, the survival rates in TCGA data set of HCC were assessed. HCC patients were assigned to two groups based upon their miR-148a-3p/5p expression levels using the minimum *P-*value approach, which is a comprehensive method to identify the optimal risk separation cutoff point in continuous gene expression measurements for survival analysis.^22^ The cutoff values for high and low miR-148a-3p and 5p expression groups were 88639 and 97.73 per million in TCGA data set, respectively. In HCC patients, those with low expression of miR-148a3p/5p had significantly worse survival than those with high miR-148a-3p/5p expression (Figure 1g). These findings suggest that decreased miR-148a-3p/5p expression is involved in the pathogenesis of HCC.

### miR-148a inhibits tumor growth and lung colonization

We investigated whether miR-148a inhibits HCC progression as suggested by its patterns of expression in human HCC patients with different survival statistics. Because FHCC98 and HepG2 HCC cell lines show high and low levels of miR-148a-3p expression, respectively, FHCC98 cells stably expressing forced miR-148a-3p sponge and HepG2 cells stably expressing Pri-miR-148a were established. Inhibition and overexpression of miR-148a-3p were confirmed by RT-qPCR (Supplementary Figure 2a). To investigate the role of miR-148a on tumor growth, an orthotopic liver cancer model was used. As shown in Supplementary Figure 2b and c, the tumors formed by miR-148a-3p sponge-expressing FHCC98 cells were bigger than those formed by control FHCC98 cells. In contrast, tumors formed by Pri-miR-148a expressing HepG2 cells were significantly smaller than the tumors formed by control HepG2 cells. To assess whether miR-148a also affects lung colonization, we directly injected the above cell lines via tail vein into nude mice and measured lung colonization 8 weeks later. As shown in Supplementary Figure 2d–f, miR-148a-3p inhibition in FHCC98 cells increased lung colonization whereas Pri-miR-148a overexpression in HepG2 cells significantly decreased lung colonization incidence. These results suggest that miR-148a inhibited both tumor growth and lung colonization.

### miR-148a deletion promotes DEN-induced hepatocarcinogenesis in mice

**Figure 2:**
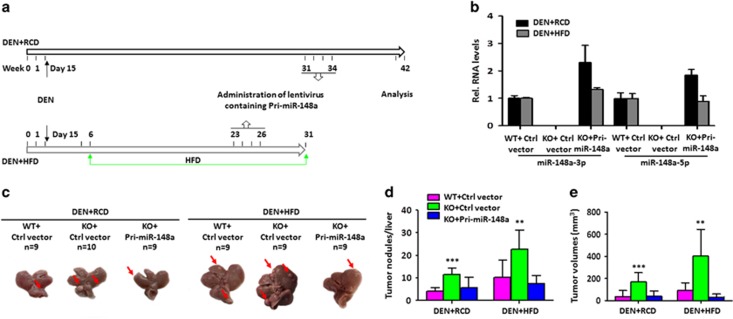
MiR-148a deletion enhances DEN-induced hepatocarcinogenesis in mice. (**a**) Schematic overview of lentivirus containing Pri-miR-148a or control vector administration during DEN-induced hepatocarcinogenesis in mice fed with RCD or HFD. (**b**) RT-qPCR detection miR-148a-3p/5p RNA levels in tumors derived from (**a**). (**c**) Representative images of tumor nodules in the livers of mice (*n*=10). (**d**) Quantitation of tumor nodules in the liver of mice. (**e**) Tumor volumes in the liver of mice. Data present mean±S.D. in (**b**), (**d**) and (**e**). **P<0.01; ***P<0.001

To determine the effect of miR-148a deletion on hepatocarcinogenesis, a DEN-induced mouse HCC model was used. Wild type (WT) and miR-148a knockout (KO) male mice were treated with DEN at postnatal day 15 on either RCD or HFD (Figure 2a). Then miR-148a KO mice were randomly divided into two groups. One group was treated with concentrated pHAGE-Pri-miR-148a lentivirus and the other group was treated with concentrated pHAGE-GFP lentivirus (Figure 2a). Strikingly, the number of tumor nodules per liver were significantly higher in miR-148a KO mice than in WT mice (Figure 2b–d) and tumor sizes were bigger (Figure 2e) irrespective of the animals’ diet. Moreover, restoring Pri-miR-148a expression reduced the size of tumor nodules and also tumor growth rates (Figure 2b–e) in miR-148a KO liver. These findings suggested that miR-148a deletion promotes hepatic tumor progression in mice.

### miR-148a deficiency enhances hepatic steatosis

**Figure 3:**
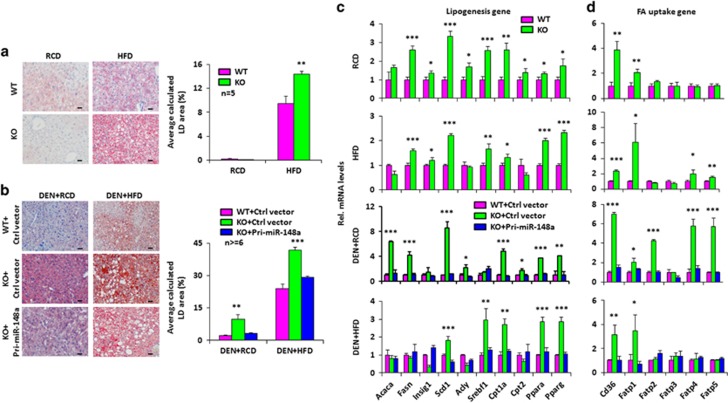
Loss of miR-148a causes hepatic lipid accumulation. (**a** and **b**) Left: Representative images of ORO-stained liver histological sections from the WT and miR-148a KO mice fed a RCD or HFD with or without DEN treatment. Right: Quantifications of the lipid droplets (LDs) volumes from the areas of ORO-stained liver sections. (**c-d**) RT-qPCR analysis of the RNA levels of hepatic genes involved in lipogenesis (**c**) and fatty acid (FA) uptake (**d**) in the mice from (**a** and **b**). Data present mean±S.D. *P<0.05; **P<0.01; ***P<0.001

Excessive lipid anabolism and storage caused by deregulation of lipid and cholesterol metabolism are now increasingly recognized to promote hepatocarcinogenesis.^2, 23^ Given that miR-148a deficiency also enhances hepatocarcinogenesis, hepatic steatosis in these animals was assessed by quantification of hepatic neutral lipid by ORO staining in hepatic tissues. As shown in Figure 3a and b, miR-148a KO mice exhibited significantly increased hepatic steatosis as indicated by lipid accumulation. The expression of genes involved in lipogenesis and fatty acid uptake^24^ were also significantly upregulated in miR-148a KO mice (Figure 3c and d).

**Figure 4:**
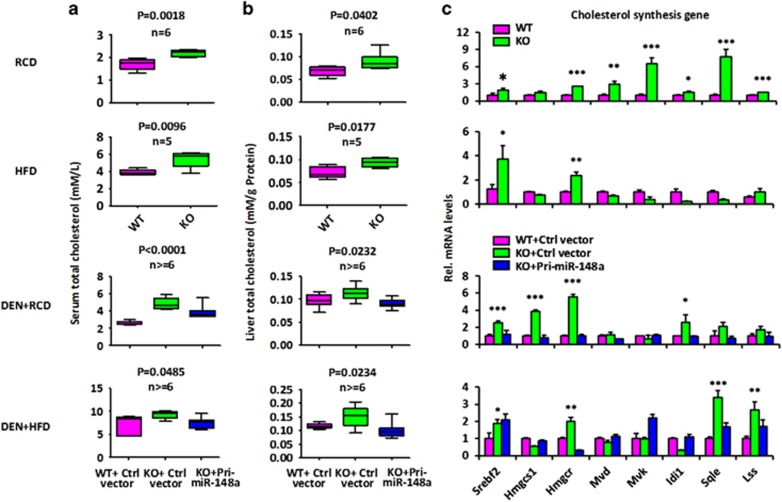
miR-148a deletion enhances cholesterol biosynthesis. (**a** and **b**) Serum (**a**) and hepatic (**b**) TC in the WT and miR-148a KO mice under RCD or HFD with or without DEN treatment (*n*=5–7). Significance was performed using Wilcoxon signed rank test. The horizontal lines in the box plots represent the median, the boxes represent the interquartile range, and the whiskers represent the minimal and maximal values. (**c**) RT-qPCR analysis of the expression of hepatic genes involved in cholesterol biosynthesis in the mice from (**a**). Data present mean±S.D. in (**c**). *P<0.05; **P<0.01; ***P<0.001

The profiles of other lipid metabolites in the serum and liver from WT and miR-148a KO mice were also measured. Regardless of the diet, miR-148a deletion led to significant increases in serum and hepatic total cholesterol (TC) without significantly altering triglyceride (TG) levels (Figure 4a and b,Supplementary Table 2). Similarly, DEN-treated miR-148a KO mice also exhibited significantly elevated serum and hepatic TC without significantly changing TG levels on either RCD or HFD (Figure 2a and Figure 4a and b,Supplementary Table 2). As hepatic and serum TC levels were increased without a significant change in TG levels in miR-148a KO mice, the increase of TC levels could be the result of increased TC biosynthesis in miR-148a KO mice. Therefore, the expression of cholesterol biosynthetic genes was measured by RT-qPCR. The results showed that most genes in this pathway were more highly expressed in miR-148a KO animals, including those encoding sterol regulatory element binding transcription factor 2 (Srebf2) and the rate-limiting enzyme, 3-hydroxy-3-methylglutaryl-CoA reductase (Hmgcr; Figure 4c). These findings strongly support that miR-148a deficiency increases cholesterol biosynthesis.

### miR-148a targets Hmgcr, Pgc1*α*, Sirt7 and Ybx1

As described above, miR-148a deletion enhances lipid metabolism and hepatocarcinogenesis. To identify potential targets of miR-148a-3p/5p, database prediction combined with biological function analyses were performed on candidate target genes involved in lipid metabolism and hepatocarcinogenesis. Pparg co-activator 1 alpha (Pgc1*α*), sirtuin 7 (Sirt7) and Hmgcr, Y-box binding protein 1 (Ybx1) which are involved in the processes of lipid metabolism and hepatocarcinogenesis were all identified as potential targets of miR-148-3p and 5p, respectively.^25, 26, 27, 28, 29^ Pgc1*α* is a transcription co-activator that regulates numerous genes involved in lipid and energy metabolism.^25^ Sirt7 controls lipid metabolism in liver by regulating the ubiquitin-proteasome pathway.^26^ Hmgcr is the rate-limiting enzyme for cholesterol synthesis.^27^ Ybx1 is a DNA and RNA binding protein and has been implicated in numerous cellular processes including regulation of transcription and translation, pre-mRNA splicing, DNA repair and mRNA packaging.^28, 29^

**Figure 5:**
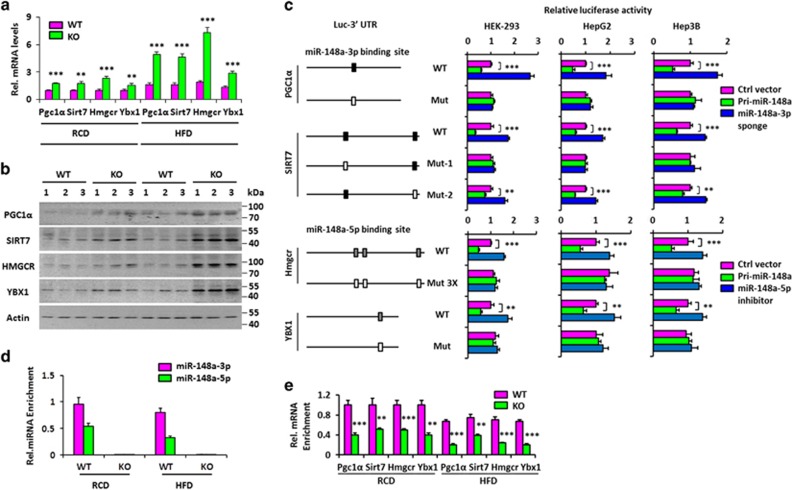
Pgc1*α*, Sirt7 and Hmgcr, Ybx1 are direct targets of miR-148a-3p and 5p, respectively. (**a**) RT-qPCR analysis of the expression of the predicted miR-148a-3p/5p targets in the hepatic tissues from the WT and miR-148a KO mice under RCD or HFD. (**b**) Western blot of the predicted miR-148a-3p/5p targets in the hepatic tissues from (**a**). (**c**) Luciferase activity of the reporter vector containing the WT or miR-148a-3p/5p-binding mutant 3′UTRs of the above predicted miR-148a-3p/5p targets was determined after co-transfection with Pri-miR-148a, miR-148a-3p sponge expression, miR-148a-5p inhibitor or control vectors in HEK293, HepG2 and Hep3B cells. (**d** and **e**) RT-qPCR analysis was performed to quantify the miR-148a-3p/5p (**d**) and their predicted targets RNA (**e**) levels incorporated into RISC in hepatic tissues from WT and miR-148a KO mice under RCD or HFD. Data present mean±S.D. in (**a**, **c**, **d** and **e**). **P<0.01; ***P<0.001

To study the effect of miR-148a-3p/5p on endogenous expression of these targets, their mRNA and protein levels in the livers of WT and miR-148a KO mice were measured and found to be elevated in the latter group (Figure 5a and b).To determine whether these targets represent direct targets of miR-148a-3p/5p, luciferase reporter assays were conducted to examine whether the putative miR-148a-binding sites in the 3′UTR of these targets were important for miR-148a-mediated suppression. Indeed, enforced miR-148 expression inhibited the activity of these targets 3′UTR reporter vector in dual luciferase reporter assays, while mutation in miR-148a binding sites abrogated this repression; Suppression of miR-148a-3p and 5p using their respective sponge or inhibitor enhanced the activity of Pgc1*α*, Sirt7 and HMGCR, YBX1 3′-UTR reporter vector in the same assays,respectively, while mutation in miR-148a-3p/5p-binding sites abrogated this upregulation (Figure 5c). These results suggested that the mRNAs of these targets are directly regulated by miR-148a-3p/5p via seeding-matching sequences.

Finally, we used an antibody against AGO-2 to co-immunoprecipitate RNAs associated with the RNA-induced Silencing Complex (RISC). As shown in Figure 5d and e, the above target RNAs were significantly enriched in WT liver extracts versus those from miR-148a KO mice. Taken together, these data indicated that miR-148a-3p/5p directly targets these molecules.

## Discussion

In this study, the intrinsic physiological function of miR-148a was investigated using mice with germline deletion of the miR-148a locus. miR-148a is downregulated in HCCs and is associated with poor prognosis as we and others have shown previously. Many studies including ours have shown that miR-148a performs a tumor suppressor function in human HCCs.^13, 14, 15, 16, 17, 18, 19, 20^ In cultured HCC cells and mouse xenograft models, miR-148a suppressed growth, epithelial-to-mesenchymal transition, invasion and metastasis through inhibition of different oncoprotein signaling pathways.^13, 14, 15, 16, 17, 18, 19, 20^ In the current study, miR-148a exerted an intrinsic tumor suppressor effect as shown by several pieces of evidence. First, miR-148a deficiency promoted DEN-induced hepatocarcinogenesis through multiple complex mechanisms in mice and the re-expression of miR-148a via lentivirus infection reversed this tumor susceptibility. This is likely mediated by the ability of miR-148a to directly and indirectly inhibit the expression of multiple, functionally related genes, which encode key factors that promote HCC progression such as c-Myc, Dnmt1, Wnt1, Ybx1, Sirt7 and Pgc1*α*. Restoration of miR-148a expression is sufficient to inhibit DEN-induced hepatocarcinogenesis in mice. Recently it has been shown that miR-148a-mimetic treatment in Pten null mice markedly suppressed tumor development and growth rates.^19^ These effects were associated with tumor cell differentiation and were at least partially mediated by IkB kinase alpha/NUMB/NOTCH signaling.^19^ Therefore, miR-148a may represent a promising candidate for miRNA replacement therapy in HCC patients.

Our study also revealed that genetic deletion of miR-148a results in hepatic lipid accumulation and increased serum and liver TC levels. These abnormalities correlated with the upregulation of several key genes whose encoded products catalyze lipogenesis and cholesterol biosynthesis. These include Hmgcr the rate-limiting enzyme of cholesterol biosynthesis and a direct miR-148a target, and other enzymes downstream of Hmgcr. miR-148a deletion also resulted in increased expression of several key factors involved in hepatic lipid metabolic transcriptional regulation, including Pgc1*α* and Sirt7. Therefore, miR-148a KO mice have abnormal hepatic lipid metabolism. Patients with hepatitis or cirrhosis also tend to have high levels of TC as well as a greatly increased risk of developing HCC.^2, 23^

In summary, we show that miR-148a deletion exerts crucial roles on lipid metabolism and hepatocarcinogenesis. These data further identify miR-148a as a potential therapeutic target for certain liver diseases, including cancer.

## Materials and methods

### Materials

DEN (73861) and HFD (D12492) were ordered from Sigma-Aldrich and Research Diets, respectively. Genomic DNA from mouse tails was isolated using the Wizard Genomic DNA Purification Kit (A1120) (Promega, Madison, WI, USA). miR-148a-5p inhibitor was purchased from RiboBio Co., Ltd. (Guangzhou, China). All other analytical grade reagents were ordered from commercial sources. Human Pri-miR-148a, ~400 bp stem-loop structures, was amplified from genomic DNA and inserted into the lentiviral vector pHAGE-CMV-GFP. miR-148a-3p sponge vector containing ten repeats of anti-sense miR-148a-3p was designed following the principles previously described,^30^ synthesized by GENEWIZ (Suzhou, China) and then cloned into the lentiviral vector pHAGE-CMV-GFP.

### Human HCC specimens

HCC samples were described previously^20, 21^ and obtained from patients undergoing tumor resection. Informed consent was obtained at the Union Hospital (Wuhan, China). The diagnosis of samples was confirmed in each case by histological review.

### DEN-induced mouse HCC model

Conventional miR-148a KO mice were ordered from Model Animal Research Center of Nanjing University (Nanjing, China). For DEN-induced mouse HCC, 15 day old WT or miR-148a KO mice were intraperitoneally injected once with 25 mg/kg DEN (Sigmal-Aldrich, St. Louis, MO, USA) and the mice were checked for development of HCC.^31^

### Lentiviral production

Viral production and transduction were described previously.^32^ In brief, viral production was performed using calcium-phosphate-mediated transfection of HEK293 cells. Virus was concentrated by ultracentrifugation. The indicated cells were infected with 10^6^ viral transducing units per ml plus polybrene and stably transduced cells were selected in puromycin for 10 days or sorted by FACS. In order to obtain high-titer vector stocks for *in vivo* experiments, the virus was ultracentrifuged to obtain a concentration of 10^8^ particles per ml. A total of 10^7^ particles were injected in PBS directly into tumor xenografts or intravenously via the lateral tail veins.

### RT-qPCR

Total RNA was isolated using Trizol reagent (Invitrogen, Carlsbad, CA, USA). Stem-loop RT for miRNAs was performed according to the protocols recommended by the manufacturer. For RT, total RNA was transcribed into cDNA using random primers and reverse transcriptase (Promega, Madcon, WI, USA). Other reagents for RT were ordered from Promega and RiboBio Co. Ltd. Quantitative PCR (qPCR) was performed with SYBRGreen (Bio-Rad, Hercules, CA, USA). U6 RNA and GAPDH were used as an internal control for miRNAs and mRNA, respectively. qPCR Primers are listed in Supplementary Table 1.

### Serum and hepatic tissue assays

The hepatic and serum TC,TG levels were determined using ELISA assay kits in accordance with the manufacturer’s instructions (Jiancheng Bio., Nanjing, China).

### Histology and immunohistochemistry

Sections from liver and lung in the above mouse models were fixed, embedded, sectioned, and stained with H&E according to the standard protocol. Ki-67 staining was performed according to the manufacturer’s protocol (mouse monoclonal antibody, 1 : 500, Sc-25280, Santa Cruz Biotechnology, Dallas, TX, USA). For apoptosis detection, Caspase-3staining was performed according to the manufacturer’s instruction (Rabbit polyclonal antibody, 1:1000, ab4051, Abcam, Cambridge, MA, USA). Oil-red-O (ORO)-staining was performed according to the standard protocol.^24, 33^

### Dual luciferase assays

Luciferase reporter assays were performed as described previously.^21, 31^ The targets of miR-148a 3′-UTRs were amplified from cDNAs, inserted into pGL3-basic vector under the control of the HSV-TK promoter. Mutations in the miR-148a seed-matching sequences were generated by overlap extension PCR. For luciferase assays, HEK293, HepG2 and Hep3B cells were transfected with the indicated firefly luciferase reporter plasmid, Renilla reporter plasmid as a normalization control, and Pri-miR-148a, miR-148a-3p sponge or a control vector. Luciferase activity was measured and analyzed using the Dual Luciferase Reporter assay (Promega, Madcon, WI, USA).

### Protein assays

Hepatic tissues and cell pellets were lysed, sonicated, quantified and resolved by SDS-PAGE. Proteins were transferred to polyvinylidene difluoride membranes which were then probed with an anti-YBX1 monoclonal antibody (1:1000) (ab76149, Abcam), or with a 1:1,000 dilution of anti-HMGCR (A1633, Abclonal, Woburn, MA, USA) poly-antibody, or anti-PGC1*α* (20658-1-AP, Proteintech, Wuhan, Hubei, China) poly-antibody, or anti-SIRT7 (12994-1-AP, Abclonal) poly-antibody as described. The blot was then incubated with a 1 : 5,000 dilution of HRP-conjugated goat anti-mouse IgG (Santa Cruz Biotechnology), washed, and subjected to chemiluminescence detection as described.^20, 21^

### Bioinformatics and statistical analyses

Online databases Targetscan (http://www.targetscan.org/), miRDB (http://www.miRdb.org/), miRwalk (http://www.ma.uni-heidelberg.de/apps/zmf/miRwalk/), PICTAR5 (http://pictar.mdc-berlin.de/) and miRanda (http://www.miRanda-im.org/) were used to predict miR-148a targets.

TCGA data analysis was performed as described previously.^34, 35^ HCC datasets were downloaded from the Cancer Genome Atlas (TCGA) data portal (http://tcga-data.nci.nih.gov).^34, 35^ MiR-148a expression data were assessed and Kaplan–Meier curves were analyzed in human HCC tissues from the TCGA miR-seq data set (*n*=355). HCC samples were assigned to two groups based on miR-148a-3p/5p expression level using the minimum *P*-value approach.^22^ Significance within the high and low expression groups was calculated using a two-sided *t*-test.

Data were expressed as the mean±S.D. or S.E.M. Statistical analysis was performed using the unpaired, two-tailed Student's *t*-test, Wilcoxon signed rank test, or Mann–Whitney *U*-test and *P*<0.05 was considered statistically significant.^20, 21^
