# Supplementary material for: MicroRNA-148a deficiency promotes hepatic lipid metabolism and hepatocarcinogenesis in mice
Source: Cell Death Dis. 2017 Jul 13;8(7):e2916–. doi: 10.1038/cddis.2017.309 (PMC5550856; doi:10.1038/cddis.2017.309)
Supplement: Supplementary Materials [file cddis2017309x1.doc]

**Supplementary materials**

**MicroRNA-148a deficiency promotes hepatic lipid metabolism and hepatocarcinogenesis in mice**

Li Cheng**^# ,1,2^**, Yahui Zhu**^# ,1,2^**, Han Han**^1,2^**, Qiang Zhang**^1,2^**, Kaisa Cui**^1,2^**, Hongxing Shen**^1,2^**, Jinxiang Zhang**^3^**, Jun Yan**^4,5^**, Edward Prochownik**^6^** and Youjun Li**^* ,1,2^**

**^#^**These authors contributed equally to this work;

**^1^**Hubei Key Laboratory of Cell Homeostasis, College of Life Sciences, Wuhan University, Wuhan, China, 430072；**^2^**Medical Research Institute, School of Medicine, Wuhan University, Wuhan, China, 430071；**^3^**Department of Surgery, Wuhan Union Hospital, Wuhan, China, 430022； **^4^**State Key Laboratory of Pharmaceutical Biotechnology and MOE Key Laboratory of Model Animals for Disease Study, Model Animal Research Center, Nanjing University, Nanjing, China, 210008；**^5^**Collaborative Innovation Center for Genetics and Development, Shanghai, China, 200438；**^6^**Division of Hematology/Oncology, Children's Hospital of Pittsburgh of UPMC and The Department of Microbiology and Molecular Genetics, The University of Pittsburgh Medical Center, Pittsburgh, Pennsylvania, USA, 15224.

*****Corresponding author: Youjun Li, Hubei Key Laboratory of Cell Homeostasis, College of Life Sciences, Medical Research Institute , Wuhan University, Wuhan, China, 430072; Email: liy7@whu.edu.cn; Phone: 86-27-68752050; Fax: 86-27-68752560

**Supplementary materials includes:**

2 Supplementary figures

2 Supplementary tables

Supplementary materials and methods

**Supplementary Figures**

**Supplementary Figure 1 (Related to Figure 1) miR-148a is a family of evolutionarily conserved miRNAs.** (**a**) miR-148a-3p/5p mature sequences are evolutionarily conserved from Homo sapiens to Sus scrofa. miR-148a-3p/5p seed sequences are indicated in magenta. (**b**) RT-qPCR analysis of the expression of Pri/Pre-miR-148a and miR-148a-3p/5p in normal human hepatocytes HL-7702 and four human liver tumor-derived cell lines FHCC98, HepG2, BEL-7402 and Huh7. Data present mean ± S.D.

**Supplementary Figure 2 (Related to Figure 1) miR-148a inhibits tumor growth and lung colonization.** (**a**) RT-qPCR analysis of miR-148a-3p RNA levels. The miR-148a-3p sponge and Pri-miR-148a, control expression vectors were transfected into FHCC98 and HepG2 cells, respectively and GFP cells were sorted using flow cytometry to get stable FHCC98 and HepG2 cells expressing the indicated vectors. RNA was isolated from the sorted GFP cells and used to RT-qPCR analysis. U6 was used as internal control. (**b**) Representative images of nude mouse livers after 4 weeks of intrahepatic inoculation with the indicated cells from A (n=3). Arrows indicated focal tumor nodules on the liver surface. (**c**) Tumor volumes in mouse livers from B. (**d**) Representative photos show colonization foci in the lungs of mice (n=3). 10^5^ of the indicated cells were injected via tail vein into the mice and lung nodules were enumerated 30-45 days later. (**e**) Number of tumor nodules in the lungs of mice from **d**. Data present mean ± S.D. in **a**, **c** and **e**. ***P*<0.01, ****P*<0.001. (**f**) Colonization nodules in lungs of mice from **d** were analyzed for H&E and PCNA staining. Scale bar, 50 *μ*m.

**Supplementary Tables**

**Supplementary Table 1** List of primer sequences used in this study.

Primers for genotyping miR-148a KO mouse.

|  | Forward Primer(5’>3’ ) | Reverse Primer(5’>3’) |
| --- | --- | --- |
| Primer 1 | AGCCACACCCATAAACATCA | AAGGGTTATTGAATATGATCGGA |
| Primer 2 | GCATCGCATTGTCTGAGTAGGTG | CCCTCACCAACTTTCCAGAT |
| Primer 3 | AGCCACACCCATAAACATCA | GTGCCAAAGCCACAGGTAATGT |

qPCR primers

| Gene | Forward Primer(5’>3’ ) | Reverse Primer(5’>3’) |
| --- | --- | --- |
| Pri-miR-148a | GAGGCAAAGTTCTGAGACAC | GTTCTGTAGTGCACTGAC |
| Pre-miR-148a | GAGACACTCCGACTCTGAGT | GTTCTGTAGTGCACTGAC |
| β-Actin | ATCATGAAGTGTGACGTGGACAT | AGGAGCAATGATCTTGATCTTCA |
| Acaca | AATGAACGTGCAATCCGATTTG | ACTCCACATTTGCGTAATTGTTG |
| Fasn | AGAGATCCCGAGACGCTTCT | GCTTGGTCCTTTGAAGTCGAAGA |
| Insig1 | CTAGTGCTCTTCTCATTTGGCG | AGGGATACAGTAAACCGACAACA |
| Scd1 | CTCCAGTTCTTACACGACCA | GACGGATGTCTTCTTCCAG |
| Acly | AATCCTGGCTAAAACCTCGCC | GCATAGATGCACACGTAGAACT |
| Srebf1 | CAAGGCCATCGACTACATCCG | CACCACTTCGGGTTTCATGC |
| Cpt1a | CTATGCGCTACTCGCTGAAGG | GGCTTTCGACCCGAGAAGA |
| Cpt2 | CCTGCTCGCTCAGGATAAACA | GTGTCTTCAGAAACCGCACTG |
| Ppara | AACATCGAGTGTCGAATATGTGG | CCGAATAGTTCGCCGAAAGAA |
| Pparg | CTCCAAGAATACCAAAGTGCGA | GCCTGATGCTTTATCCCCACA |
| Cd36 | AGATGACGTGGCAAAGAACAG | CCTTGGCTAGATAACGAACTC |
| Fatp1 | CTGGGACTTCCGTGGACCT | TCTTGCAGACGATACGCAGAA |
| Fatp2 | TCGTGGAGGTCTGAAGTCACT | GATGGTTGCCGCTTTTGGAA |
| Fatp3 | CATTGGGGAGTTGTGCCGATA | GCCAAGCGCACCTTATGGT |
| Fatp4 | TGGAAAACCGCAATGAGTTTGT | TCCCGCCTAAGGTTGGTGT |
| Fatp5 | GCAGCATGGGTCCTGAAAG | ACGGGAGAACTAAGATAGCAGC |
| Srebf2 | GCAGCGACCAGCTTTCAAGT | CTGAGGTTGCACCAGGACC |
| Hmgcs1 | GGAAATGCCAGACCTACAGGTG | CTCGGAGAGCATGTCAGGCT |
| Hmgcr | AATTCACAGGATGAAGTAAGGGA | TGACATGCAGCCGAAGCAGCACAT |
| Mvd | AGCTAGTCCACCGCTTCAACA | CAAACTCAGCCACAGTGTCCTC |
| Mvk | GGACTGCACAGCAAGCTGAC | CAGGGCTTGTCGGACAGGGT |
| Idi1 | GCTCCTGTTACAGCAGAGATCAG | GCTTCACACCAATGGCGTT |
| Sqle | GGCAGAGCCCAATGTAAAGT | CATAAGGAAGCCAACGAAGT |
| Lss | GCACACCACAGACCTGAGTTTC | CAGTGTGCTGAAGGAGAAACCAC |
| Pgc1α | CGATGACCCTCCTCACACCAA | AGATAAAGTTGTTGGTTTGGC |
| Sirt7 | TGTGATGACCCGGAGGAGCTGCGG | CCTTTCTGAAGCAGTGTCCATACT |
| Ybx1 | CCTTCGCAGTGTAGGCGATG | TCAGGAGCGCTTTCCGATCC |
| Abca1 | CGTTTCCGGGAAGTGTCCTAA | ACAGGTCCTTTAAGTGGTCA |
| Ldlr | AGTGGCGTCAGTGACAGTGT | CTGGTAGACTGGGTTGTCAA |
| Dnmt1 | GAGGCGGAAATCAAAGGAGGA | GGGAGTCTCTGGAGCTACCT |
| Myc | CCCTATTTCATCTGCGACGAG | GAGAAGGACGTAGCGACCG |

Primers for construction

| Insert | Forward Primer(5’>3’ ) | Reverse Primer(5’>3’) |
| --- | --- | --- |
| pHAGE-Pri-miR-148a | CGCGGATCCGTCGCATCCTGAACTAAATTG | CGCCTCGAGGGGAAAGGCGCAGCACGTG |
| PGC1α-3’UTR | CGCGGATCCCAACAGAACTGTCACAGCTT | CGCTCTAGACTTTCTGAGGAGCGGACACC |
| PGC1α-3’UTR –Mut | ACAGCTACTGAGCGTAAATGCAGCCT | GCATTTACGCTCAGTAGCTGTAACA |
| SIRT7-3’UTR | CGCTCTAGATCACGTGCTCGATGAAGAAC | CGCGGATCCGCCAGTGCAGAAACGTTTA |
| SIRT7-3’UTR-Mut-1 | AAGCCCCTCTGAGCGCTGCGGTTGTACCCTG | AACCGCAGCGCTCAGAGGGGCTTCCTCA |
| SIRT7-3’UTR-Mut-2 | CGCTCTAGATCACGTGCTCGATGAAGAAC | CGCGGATCCGCCGCTCAGGAAACGTTTAAT |
| Hmgcr-3’UTR | CGCGGATCCTCCTCAGATGTGGGAACTCT | CGCGGATCCCTGTTCCCACACTCTAAGTTC |
| Hmgcr-3’UTR-Mut-1 | GTAGCTGACGCTGACGCTGATCTTTATTTATTCAG | AAGATCAGCGTCAGCGTCAGCTACAGTGTCATTT |
| Hmgcr-3’UTR-Mut-2 | GGGTTGTGCTGACGTGCAATCTAAGTTAT | GATTGCACGTCAGCACAACCCAGACTGAATAAAT |
| Hmgcr-3’UTR-Mut-3 | CGCGGATCCTCCTCAGATGTGGGAACTCT | CGCGGATCCCGTCAGCGAGAGGTCCGACTTGCTTGTA |
| YBX1-3’UTR | CGCTCTAGAATGCCGGCTTACCATCTCTACC | CGCGGATCCGACCTTTATTAACAGGTGCTTGC |
| YBX1-3’UTR-Mut | GAGATTTTTAGCTGACGCATTTTTAATTTG | TAAAAATGCGTCAGCTAAAAATCTCAAC |

Sequence for miR-148a-3p sponge construction (5’>3’)

CGCGGATCCACAAAGTTCCATGTGCACTGAACAAAGTTCCATGTGCACTGAACAAAGTTCCATGTGCACTGAACAAAGTTCCATGTGCACTGAACAAAGTTCCATGTGCACTGAACAAAGTTCCATGTGCACTGAACAAAGTTCCATGTGCACTGAACAAAGTTCCATGTGCACTGAACAAAGTTCCATGTGCACTGAACAAAGTTCCATGTGCACTGACTCGAGGCG

**Supplementary Table 2**

Serum TG, TC, ALT, AST, glucose levels and hepatic TG, TC levels in the WT and miR-148a KO mice under RCD or HFD with or without DEN treatment.

| RCD | | WT | miR-148a KO | |
| --- | --- | --- | --- | --- |
|  |  | (Mean ± S.D.) | (Mean ± S.D.) | p-value |
| Serum | TG(mM/L) | 0.655±0.238 | 0.757±0.226 | 0.4647 |
|  | TC(mM/L) | 1.710±0.250 | 2.208±0.147 | 0.0018 |
|  | ALT(U/L) | 10.708±3.996 | 8.172±1.441 | 0.2187 |
|  | AST(U/L) | 12.302±2.853 | 10.315±3.666 | 0.3669 |
|  | Glucose(mM/L) | 10.321±5.875 | 11.239±4.722 | 0.7698 |
| Liver | TG(mM/g) | 0.178±0.040 | 0.214±0.039 | 0.1452 |
|  | TC(mM/g) | 0.068±0.011 | 0.089±0.019 | 0.0402 |

| HFD | | WT | miR-148a KO | |
| --- | --- | --- | --- | --- |
|  |  | (Mean ± S.D.) | (Mean ± S.D.) | p-value |
| Serum | TG(mM/L) | 1.034±0.212 | 0.860±0.382 | 0.3977 |
|  | TC(mM/L) | 3.878±0.311 | 5.437±0.981 | 0.0096 |
|  | ALT(U/L) | 21.134±15.059 | 21.270±6.683 | 0.9858 |
|  | AST(U/L) | 15.948±7.025 | 13.921±3.814 | 0.5861 |
|  | Glucose(mM/L) | 10.321±5.875 | 11.239±4.722 | 0.7698 |
| Liver | TG(mM/g) | 0.164±0.046 | 0.209±0.075 | 0.2896 |
|  | TC(mM/g) | 0.072±0.012 | 0.093±0.010 | 0.0177 |

| DEN+RCD | | WT+Ctrl vector | KO+Ctrl vector | | KO+Pri-miR-148a | |
| --- | --- | --- | --- | --- | --- | --- |
|  |  | (Mean ± S.D.) | (Mean ± S.D.) | p-value | (Mean ± S.D.) | p-value |
| Serum | TG(mM/L) | 0.804±0.284 | 0.776±0.314 | 0.8466 | 0.819±0.272 | 0.7742 |
|  | TC(mM/L) | 2.582±0.208 | 4.799±0.648 | <0.0001 | 3.892±0.782 | 0.0359 |
|  | ALT(U/L) | 11.811±2.373 | 17.791±3.084 | 0.0005 | 13.854±3.637 | 0.0417 |
|  | AST(U/L) | 19.906±2.991 | 23.912±3.232 | 0.0331 | 14.848±3.182 | 0.0002 |
|  | Glucose(mM/L) | 13.938±2.428 | 11.850±3.290 | 0.1616 | 12.302±6.527 | 0.8585 |
| Liver | TG(mM/g) | 0.260±0.064 | 0.425±0.127 | 0.0018 | 0.225±0.086 | 0.0010 |
|  | TC(mM/g) | 0.097±0.014 | 0.113±0.014 | 0.0232 | 0.091±0.009 | 0.0011 |

| DEN+HFD | | WT+Ctrl vector | KO+Ctrl vector | | KO+Pri-miR-148a | |
| --- | --- | --- | --- | --- | --- | --- |
|  |  | (Mean ± S.D.) | (Mean ± S.D.) | p-value | (Mean ± S.D.) | p-value |
| Serum | TG(mM/L) | 1.212±0.276 | 0.779±0.147 | 0.0012 | 1.026±0.205 | 0.0152 |
|  | TC(mM/L) | 7.253±2.007 | 9.246±0.835 | 0.0485 | 7.452±1.121 | 0.0054 |
|  | ALT(U/L) | 56.648±12.668 | 86.793±18.903 | 0.0088 | 67.107±8.431 | 0.0421 |
|  | AST(U/L) | 18.474±5.996 | 27.731±5.128 | 0.0178 | 21.525±5.251 | 0.0450 |
|  | Glucose(mM/L) | 16.979±2.759 | 16.402±5.908 | 0.7961 | 24.230±4.741 | 0.0084 |
| Liver | TG(mM/g) | 0.467±0.129 | 0.654±0.301 | 0.1292 | 0.810±0.177 | 0.2075 |
|  | TC(mM/g) | 0.117±0.010 | 0.151±0.037 | 0.0234 | 0.100±0.026 | 0.0049 |

**Supplementary materials and methods**

**Cell culture.** Normal human hepatocytes HL-7702, human HEK-293 and human HCC cells (FHCC98, HepG2, Hep3B, MHCC97L, BEL-7402 and Huh-7) cells were cultured as described previously.^1,2^

**AGO2 RNA immunoprecipitation.** RNA immunoprecipitations (RIP) were performed according to the methods described previously.^2,3^ Briefly, tissues derived from WT and miR-148a KO hepatic tissues were UV irradiated and lysed with RIP buffer containing RNase Inhibitor (EO0381, Thermo Scientific) and proteinase inhibitor (Sigma-Aldrich) and then treated with DNase I (Thermo Scientific). The separated supernatant was incubated with 1mg rabbit mono-antibody against AGO2 (D2C9, Cell Signaling) or control IgG for 4 hours and then added to protein G beads (Life Technology). After digestion of protein, the precipitated RNA was purified using Trizol reagent (Life Technology) and analyzed by RT-qPCR.

**Stable cells expressing miR-148a-3p sponge and Pri-miR-148a.** This was performed as described previously.^2,3^ To establish stable expression of miR-148a-3p sponge and Pri-miR-148a, FHCC98 and HepG2 cells were transfected with the above indicated vector for 6 hours by using Lipofectamine 2000 (Invitrogen). Stably transduced cells were selected in puromycin at least 10 days or sorted by flow cytometry. Stable cell lines were examined for the expression of miRNA by RT-qPCR.

**Orthotopic liver cancer model.** Four-week-old male BALB/c nude mice were purchased from Changsha (China) SLAC Laboratory Animal Co. and maintained in microisolator cages. Tumorigenicity assays and tumor volume measurements were performed as described previously.^2-4^ Orthotopic liver tumors were established following the principles described previously.^4^ Briefly, under sterile conditions, approximately 10^6^ indicated cells were injected into the lateral left lobe of the liver of male BALB/c nude mice. 4~6 weeks after injection, the number and tumor size of tumor nodules greater than 1 mm in diameter in hepatic tissues was counted and measured. Lung, liver and adjacent tissues were harvested for protein and RNA assays as well as standard pathological studies.

**Lung colonization assay.** Lung colonization assays were performed following the principles described previously.^4^ Briefly, 5×10^6^ indicated cells were suspended in 100 µL serum-free DMEM and injected intravenously via the lateral tail vein of nude mice. All mice were kept for about 5-8 weeks until imaged by small-animal IVIS Lumina II imaging (Life Sciences). Presence or absence of lung colonization was observed. Lung, liver, and adjacent tissues were harvested for protein and RNA assays as well as pathological studies.

**Administration of lentivirus expressing Pri-miR-148a in DEN-induced mouse HCC**. This was performed as described previously.^2,5,6^ For administration of lentivirus in the DEN-induced HCC mice model, male C57BL/6 mice were DEN-treated and at in the dicated time post treatment they were randomly distributed into 2 groups. The first group of mice (n=9~10) was treated with lentivirus expressing control vector, the second group of mice (n=9~10) was treated with lentivirus expressing Pri-miR-148a. Concentrated virus in a volume of 100ul was injected in the tail vein of the mice and the treatments were performed twice per week for 4 weeks. At indicated time, tumor burden was evaluated after sacrificing the mice. Visible tumors larger than 1 mm were counted and the tumor size was determined. Lung, liver, and adjacent tissues were harvested for RNA and protein assays as well as pathological studies.^2-6^

**Supplementary references**

1. Han H, Sun D, Li W, Shen H, Zhu Y, Li C *et al*. A c-Myc-MicroRNA functional feedback loop affects hepatocarcinogenesis. *Hepatology* 2013; **57**: 2378-2389.
2. Han H, Li W, Shen H, Zhang J, Zhu Y, Li Y. microRNA-129-5p, a c-Myc negative target, affects hepatocellular carcinoma progression by blocking the Warburg effect. *J Mol Cell Biol* 2016; **8**: 400-410.
3. Wang H, Sun T, Hu J, Zhang R, Rao Y, Wang S *et al*. miR-33a promotes glioma-initiating cell self-renewal via PKA and NOTCH pathways. *J Clin Invest* 2014; **124**: 4489-4502.
4. Yang H, Cho ME, Li TW, Peng H, Ko KS, Mato JM *et al*. MicroRNAs regulate methionine adenosyltransferase 1A expression in hepatocellular carcinoma. *J Clin Invest* 2013; **123**: 285-298.
5. [Bonci D](https://www.ncbi.nlm.nih.gov/pubmed/?term=Bonci D%5BAuthor%5D&cauthor=true&cauthor_uid=18931683), [Coppola V](https://www.ncbi.nlm.nih.gov/pubmed/?term=Coppola V%5BAuthor%5D&cauthor=true&cauthor_uid=18931683), [Musumeci M](https://www.ncbi.nlm.nih.gov/pubmed/?term=Musumeci M%5BAuthor%5D&cauthor=true&cauthor_uid=18931683), [Addario A](https://www.ncbi.nlm.nih.gov/pubmed/?term=Addario A%5BAuthor%5D&cauthor=true&cauthor_uid=18931683), [Giuffrida R](https://www.ncbi.nlm.nih.gov/pubmed/?term=Giuffrida R%5BAuthor%5D&cauthor=true&cauthor_uid=18931683), [Memeo L](https://www.ncbi.nlm.nih.gov/pubmed/?term=Memeo L%5BAuthor%5D&cauthor=true&cauthor_uid=18931683) *et al*. The miR-15a-miR-16-1 cluster controls prostate cancer by targeting multiple oncogenic activities. *Nat Med* 2008; **14**: 1271-1277.
6. [Hatziapostolou M](https://www.ncbi.nlm.nih.gov/pubmed/?term=Hatziapostolou M%5BAuthor%5D&cauthor=true&cauthor_uid=22153071), [Polytarchou C](https://www.ncbi.nlm.nih.gov/pubmed/?term=Polytarchou C%5BAuthor%5D&cauthor=true&cauthor_uid=22153071), [Aggelidou E](https://www.ncbi.nlm.nih.gov/pubmed/?term=Aggelidou E%5BAuthor%5D&cauthor=true&cauthor_uid=22153071), [Drakaki A](https://www.ncbi.nlm.nih.gov/pubmed/?term=Drakaki A%5BAuthor%5D&cauthor=true&cauthor_uid=22153071), [Poultsides GA](https://www.ncbi.nlm.nih.gov/pubmed/?term=Poultsides GA%5BAuthor%5D&cauthor=true&cauthor_uid=22153071), [Jaeger SA](https://www.ncbi.nlm.nih.gov/pubmed/?term=Jaeger SA%5BAuthor%5D&cauthor=true&cauthor_uid=22153071) *et al*. An HNF4a-miRNA inflammatory feedback circuit regulates hepatocellular oncogenesis. *Cell* 2011; **147**: 1233-1247.
