# Supplementary figures and images for: MicroRNA-148a deficiency promotes hepatic lipid metabolism and hepatocarcinogenesis in mice
Source: Cell Death Dis. 2017 Jul 13;8(7):e2916–. doi: 10.1038/cddis.2017.309 (PMC5550856; doi:10.1038/cddis.2017.309)

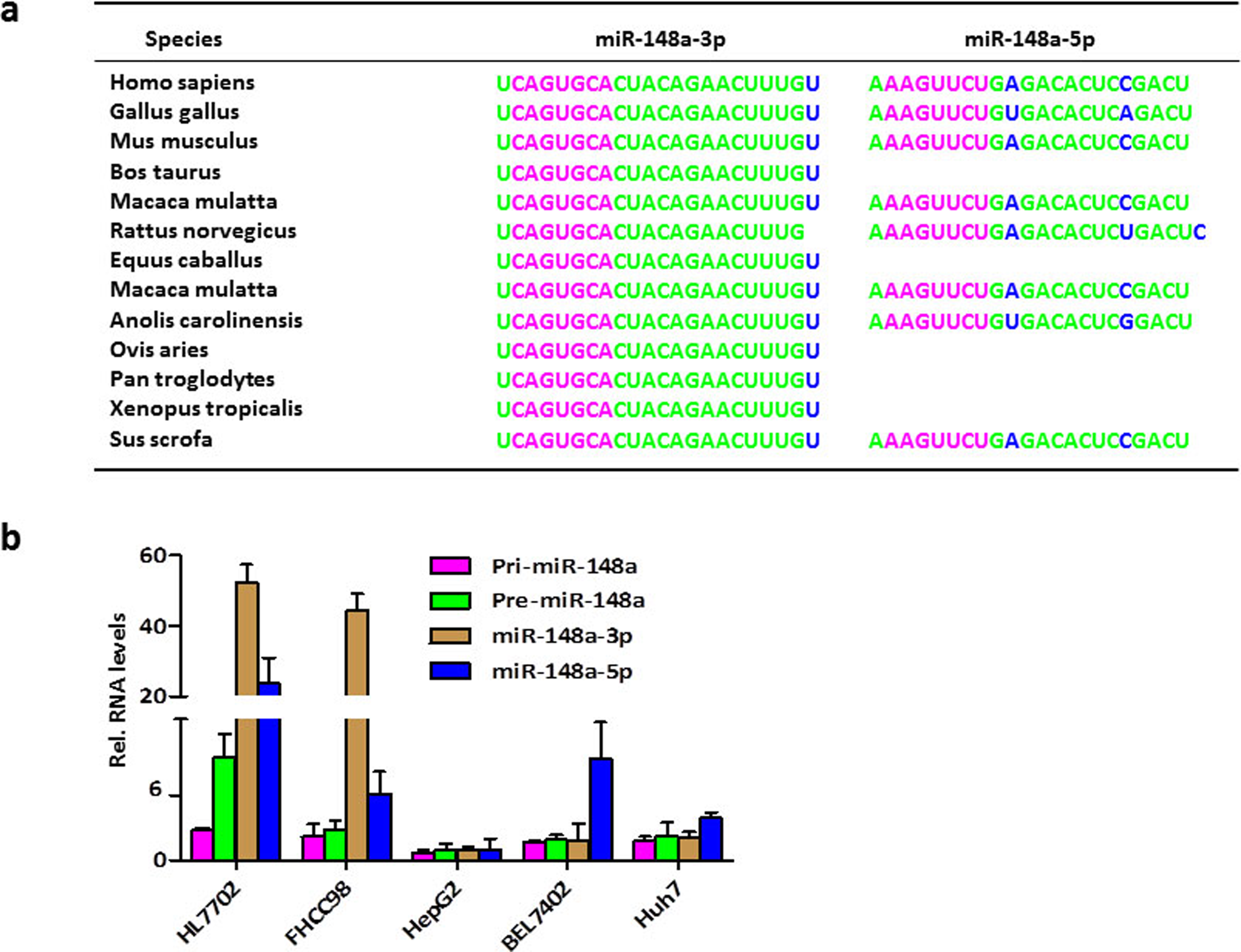

Supplement: Supplementary Figure 1 [file cddis2017309x2.tif]

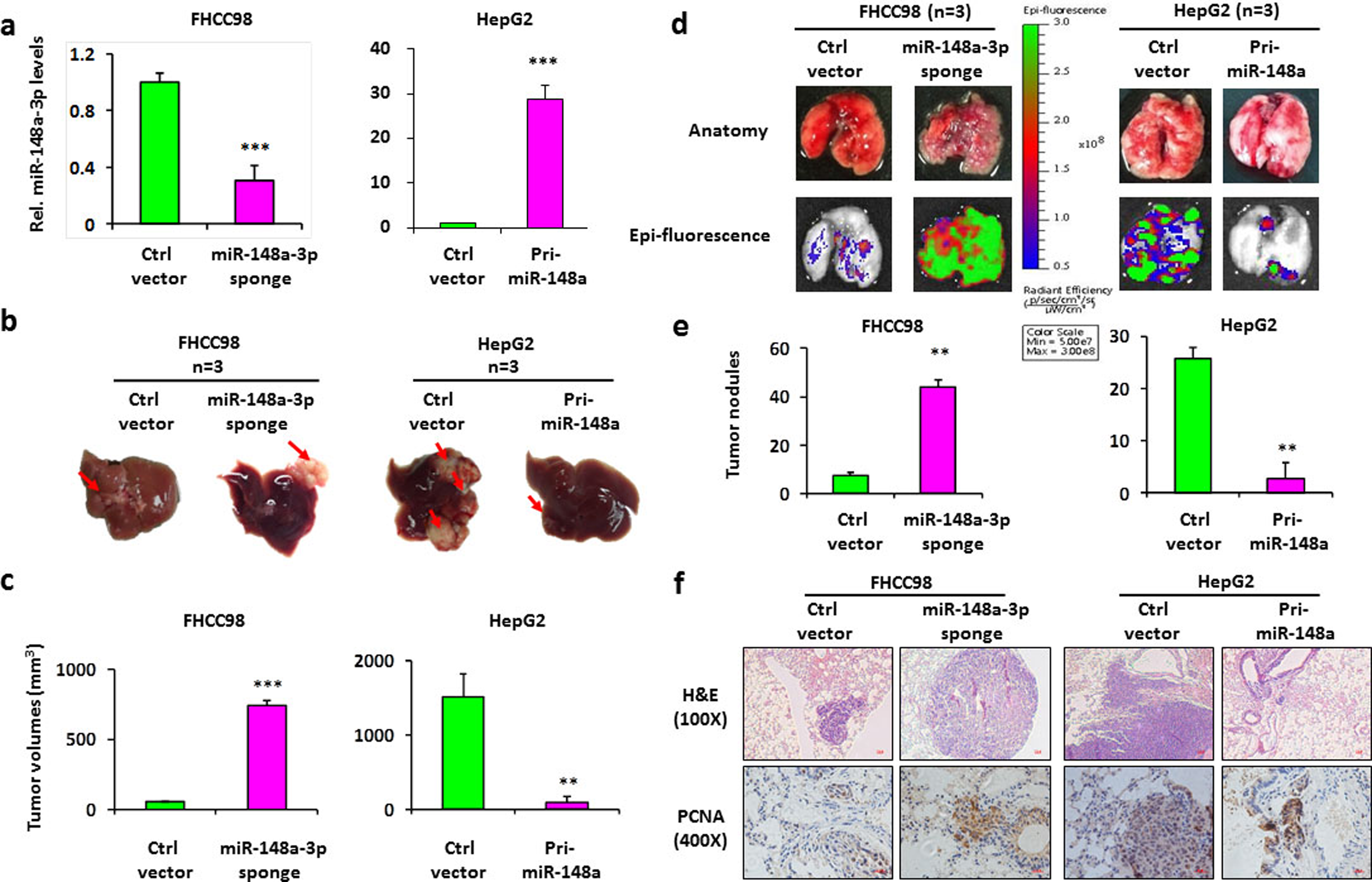

Supplement: Supplementary Figure 2 [file cddis2017309x3.tif]
